# Understanding experiences of optimal survivorship care: findings from a population-based study of cancer survivors

**DOI:** 10.1007/s00520-025-09461-2

**Published:** 2025-04-21

**Authors:** Victoria White, Karla Gough, Eli Ristevski, Karolina Lisy, Kate Webber, Jon Emery, Peter Gibbs, Maarten IJzerman, Nikki Davis, Michael Jefford

**Affiliations:** 1 School of Psychology, Faculty of Health, Deakin University, Burwood, VIC Australia; 2 Department of Health Services Research, Peter MacCallum Cancer Centre, Melbourne, VIC Australia; 3 Department of Nursing, Faculty of Medicine, Dentistry and Health Services, University of Melbourne, Parkville, VIC Australia; 4 School of Rural Health, Monash University, Warragul, VIC Australia; 5 Australian Cancer Survivorship Centre, Peter MacCallum Cancer Centre, Melbourne, VIC Australia; 6 Sir Peter MacCallum Department of Oncology, The University of Melbourne, Melbourne, VIC Australia; 7 School of Clinical Sciences, Monash University, Clayton, VIC Australia; 8 Department of Oncology, Monash Health, Clayton, VIC Australia; 9 Department of General Practice and Centre for Cancer Research, University of Melbourne, Melbourne, Australia VIC; 10 Department of Medical Oncology, Western Health, St. Albans, VIC, Australia; 11 Personalised Oncology Division, Walter and Eliza Hall Institute of Medical Research, Parkville, VIC Australia; 12 Melbourne School of Population and Global Health and Centre for Cancer Research, University of Melbourne, Melbourne, Australia VIC; 13 Primary Care Cancer Collaborative Clinical Trials Group, Victorian Comprehensive Cancer Centre, Melbourne, VIC Australia

**Keywords:** Cancer, Cancer survivors, Disparities, Satisfaction, Experience, Patient-reported outcomes, Quality of life

## Abstract

**Background:**

Multiple frameworks describing optimal cancer survivorship care recommend the development of systems to monitor delivery of quality care. This study reports the experiences of cancer survivorship care in Australia and examines associations with patient-level sociodemographic and clinical characteristics.

**Methods:**

People aged ≥ 16 years, with any cancer receiving cancer care in a Victorian public hospital in 2018, were invited to complete a survey assessing care experiences. Seven items assessed follow-up care experiences with optimal care indicated by the response: ‘Yes, definitely received’, and sub-optimal care by responses ‘Yes, I think so’, ‘No’, and ‘Not sure’. A composite score was derived with optimal care defined as positive experiences on the majority of items. Sociodemographic and clinical factors associated with optimal care were examined using multivariable logistic regression.

**Results:**

Of the 4998 (47% response rate) respondents, 3555 were receiving follow-up care. The item most respondents indicated receiving optimal care was ‘receiving information about schedule of tests/check-ups’ (73%), with optimal care least likely to be reported for the item ‘receiving information about new symptoms needing investigation’ (44%). Based on our composite measure, only 40% had optimal survivorship care overall. Those more likely to report optimal survivorship care were male, from lower socioeconomic advantage areas, reported excellent health, diagnosed with breast, prostate, lung or a haematological cancer, and diagnosed less recently.

**Conclusions:**

Large numbers of Australian cancer survivors report sub-optimal survivorship care, with experiences varying by some sociodemographic and clinical characteristics. Understanding reasons for differences can provide insight into strategies to ameliorate variations.

**Supplementary Information:**

The online version contains supplementary material available at 10.1007/s00520-025-09461-2.

## Introduction

Worldwide, the number of cancer survivors is growing [1]. Although many survivors recover well after their acute cancer care, a substantial proportion experience ongoing difficulties including living with long-term side-effects and reduced quality of life (QoL) [2]. Supportive care needs are also high which includes needs relating to managing fear of cancer recurrence [3, 4]. To assist patients to manage their recovery and monitor for recurrence and new cancers, as well as for physical and psychosocial effects, follow-up care is generally recommended, which could be with the cancer care team and/or their general practitioner [5–7]. Since the Institute of Medicine’s report on the follow-up care needs of cancer survivors [8], a growing number of organisations have developed survivorship care recommendations that incorporate a holistic approach to care. These recommendations often call for the development of survivorship care plans that include information about type and frequency of medical appointments and tests, monitoring and management of adverse events, identifying and addressing psychosocial needs, and lifestyle recommendations to promote optimal health outcomes [9, 10]. In Australia, a Survivorship Quality Care Framework has been developed that recognises the need to monitor and manage the physical, emotional, practical, and social effects of cancer and its treatment, as well as monitoring for recurrence and new cancers [5]. A recent proposal for national survivorship care standards in the US promoted the need for the health system to adopt a multidisciplinary approach to survivorship care to enable it to deliver care that focuses on meeting the survivor’s physical, emotional, and social needs [11].

A key recommendation in these different frameworks is the development of systems to monitor progress towards delivering quality survivorship care. Nekhlyudov and colleagues have proposed a number of indicators to assess quality of survivorship care including patient-reported experience measures (PREMs) assessing access to services, communication, and care coordination [6] which is in line with calls to understand patients’ experiences as part of assessing quality of care [5, 12–16]. Although numerous studies have assessed experiences of care during treatment [17–21], there is less information on survivorship care experiences with studies in this area mainly focusing on the use of survivorship care plans (see for example [22–24]). Several studies from the US have used the Medical Expenditure Panel Survey (MEPS)-Experiences with Cancer Survivorship module to examine survivorship care experiences. Data from the 2011 survey found that 62% of survivors received detailed information about monitoring and follow-up tests needed but only 29% received information about emotional/social needs [25]. Similar results were found in the 2016 MEPS [26].

Several studies have suggested that survivorship care experiences can differ by disease and sociodemographic factors, although the influence of these factors may differ between countries. Work from the US has found those from lower socioeconomic status (SES) groups, and those from minority ethnic groups are less likely to report receiving quality survivorship care or receiving detailed follow-up care information [25, 27]. In contrast, an Australian study of colorectal patients diagnosed in 2012/2013 found that those from a non-English speaking background were more likely to report receiving a written follow-up care plan, and that SES had no relationship [28]. A Canadian study involving patients with a range of cancers found that males, those who were married, those who spoke French, and those with less education were more likely to report positive follow-up experiences [29].

There is a lack of recent data regarding survivorship care experiences in Australia and elsewhere, making it difficult to know whether current survivorship care is meeting the needs of cancer survivors. To better understand the care experiences of people diagnosed with cancer in Victoria, Australia, the Victorian Department of Health commissioned a Cancer Patient Experiences Survey (CPES) which was conducted across all Victorian public hospitals in 2019. This study is aimed at interrogating the Victorian CPES dataset to assess follow-up care experiences of Victorian patients and investigating associations between follow-up experiences and selected sociodemographic and clinical characteristics.

## Methods

### Study design and setting

Analysis of data collected via a cross-sectional survey commissioned by the Victorian Department of Health and was conducted by a contracted survey administrator. This study focuses on responses from participants in follow-up care. Analysis of the data set had institutional ethics approval (HREC/76910/PMCC).

### Participants and procedure

People aged ≥ 16 years who received at least some of their cancer care (including surgery, chemotherapy, or radiotherapy) as an inpatient or outpatient in an adult Victorian public hospital in 2018 were eligible for the survey. Cancer care in Australia is delivered in the public hospital (funded by government, no costs to patients) and private hospital (funded by patient either themselves or through private health insurance) systems, with patients choosing the hospital system for each treatment (i.e. surgery, chemotherapy). Therefore, patients eligible for this survey may have had some elements of their care, including follow-up care, in the private system.

Victorian public hospitals delivering cancer care identified eligible participants and provided contact details to the survey administrator, who mailed them the survey, invitation letter, information sheet, and reply-paid envelope. The invitation letter stated that the survey was being conducted by the Victorian Department of Health. English-speaking participants could complete either an electronic or paper version of the survey. People speaking a language other than English (identified from hospital data) completed a professionally translated paper version of the survey in their preferred language.

### Variables and data sources

**Fig. 1:**
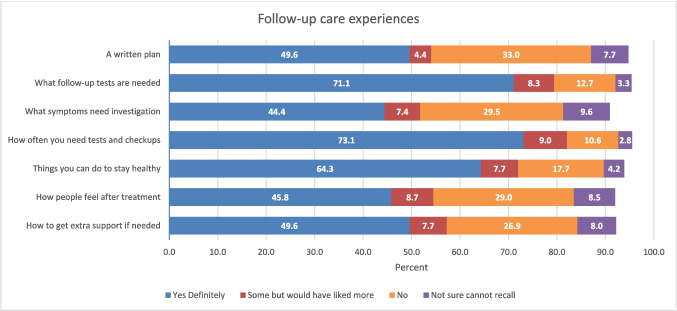
Receipt of information relating to the seven areas of survivorship care (missing responses included in analyses but data not shown, hence total does not add to 100%)

The CPES survey was based on items in the UK’s National Cancer Patient Experience Survey [30], and findings from a literature review, focus groups, and interviews with patients and health professionals, and was pilot tested at multiple health services [31, 32]. The questionnaire was divided into different care episodes, reflecting diagnosis, treatment (i.e. surgery, radiotherapy, chemotherapy), follow-up care, general experiences regarding communication with and between health professionals, care co-ordination, and information provision. Respondents completed sections relevant to their care. Most questions followed a format asking if a specific care element occurred or specific information was provided with responses generally made on a 4-point scale of ‘Yes, definitely’, ‘Yes, I think so’, ‘No, I don’t think so’, or ‘No, definitely not’. Options for not recalling and not applicable were provided. This paper utilises data from seven items that directly assess follow-up care experiences (see Fig. 1, Table 2 for items).

Demographics (see Table 1), cancer type, and location of each treatment (health service name and postcode) included for follow-up care were also assessed.

**Table 1:** Demographic and cancer characteristics of sample and by follow-up care status

Characteristic	Total^a^		Not in follow-up		In follow-up		*p*-value
	*N* = 4998		*N* = 1663		*N* = 3335		
	*n*	%	*n*	%	*n*	%	
Gender							
Male	2444	49	871	52	1573	47	
Female	2440	49	713	43	1722	52	
Missing	139	2	79	5	35	1	< 0.001
Age (in years)							
< 50	538	11	119	7	419	12	
50–59	753	15	180	11	573	17	
60–69	1450	29	434	26	1016	30	
70–79	1410	28	525	31	885	27	
≥ 80	560	11	262	16	298	9	
Missing	287	6	132	9	144	4	< 0.001
Socioeconomic disadvantage^b^							
Highest disadvantage (40%)	1577	32	538	32	1039	31	
Mid (40%)	1771	35	602	34	1169	35	
Highest advantage (20%)	778	16	232	14	546	16	
Missing	872	17	291	17	581	17	0.17
Follow-up care treatment centre location^c^							
Metropolitan area	3718	74	1217	73	2501	75	
Regional centre	1050	21	364	22	686	21	
Large/medium/small/remote towns	230	5	83	5	148	4	0.37
Language spoken at home							
Not English	476	10	149	9	327	10	
English	4522	90	1514	91	3008	90	0.34
Aboriginal or Torres Strait Islander							
Yes	36	1	10	1	26	1	
No	4962	99	1653	99	3309	99	0.48
Self-reported health status							
Excellent	545	11	155	9	390	12	
Very good/good	3122	64	957	57	2254	68	
Fair/poor	1062	21	447	27	615	18	
Missing	180	4	104	6	76	2	< 0.001
Cancer type							
Colorectal	473	9	141	8	332	10	
Breast	838	17	172	10	666	20	
Prostate	615	12	246	15	369	11	
Haematological	984	20	377	23	607	18	
Lung/Mesothelioma	327	7	144	9	183	5	
Melanoma/skin	224	4	75	5	149	4	
Gynaecological^d^	198	4	42	2	156	5	
Urological	323	6	106	6	217	7	
Other	1016	20	360	22	656	20	< 0.001
Year of diagnosis							
2015 or earlier	639	13	251	15	388	12	
2016	258	5	78	5	171	5	
2017	908	18	224	13	685	21	
2018	2724	55	880	53	1984	55	
Missing	469	9	221	13	248	7	< 0.001
Number of treatment^e^ modalities							
1	2752	55	1151	69	1601	48	
2	1345	27	342	21	1003	30	
3	694	14	137	8	557	17	
4	207	4	33	2	174	5	< 0.001
Treatment							
Surgery	2857	57	537	32	2320	70	< 0.001
Radiotherapy	1688	34	347	21	1341	40	< 0.001
Chemotherapy	2288	46	677	41	1611	48	< 0.001
Hormone blocking therapy	753	15	166	10	587	18	< 0.001
Health care system for follow-up care							
Public	2748	55	271	16	2477	74	
Private	692	14	79	5	613	18	
Missing	1558	31	1313	79	245	7	< 0.001

^a^Total respondents participating in survey

^b^Based on Australian Bureau of Statistics Index of Relative Socioeconomic Advantage and Disadvantage [33]

^c^Based on Modified Monash Model location index [34]

^d^Gynaecological includes uterus, ovarian, cervical, vulvar, vaginal, endometrial, and fallopian tube

^e^Active surveillance/watchful waiting included as a management modality

### Data handling and analysis

Analyses were undertaken using STATA (version 17) and IBM SPSS Statistics (version 27). Prior to formal analysis, descriptive statistics identified missing and out-of-range values and items with low response rates.

Following others [25], responses to follow-up care items were recoded into three categories: ‘Yes, definitely’ (2, indicating optimal care), ‘Yes, I think so’ (1), and all other responses coded (0). A composite rating was calculated by summing responses across the seven follow-up care items and calculating the mean to ensure scores remained on the 0–2 scale, with this measure used as an indicator of survivorship care overall. Following others [25, 29], this score was dichotomised to indicate overall optimal survivorship care (mean score ≥ 1.5 indicating the majority of items were scored 2) or not (sub-optimal care). Internal consistency reliability for the measure was good (Cronbach’s alpha: follow-up = 0.87).

Residential postcode was used to assign socioeconomic status to each participant via the Australian Bureau of Statistics’ Index of Relative Socioeconomic Advantage and Disadvantage. This postcode-based indicator provides a score summarising levels of employment, income, education, and other economic and social conditions of an area [33]. Quintile cutoffs are provided, and these were categorised into three groups indicating the 20% least disadvantage, the 40% most disadvantaged, and the 40% with mid-levels of disadvantage. Participants provided the name and location (town, suburb, or postcode) of the health service managing their follow-up care, and this service was assigned to the private or public hospital system and to a metropolitan, regional, rural, or remote location using the Modified Monash (MM) Model, the measure of location used by the Australian Government [34]. The MM Model classifies areas into one of seven categories ranging from metropolitan (1) to very remote (7) based on the Australian Bureau of Statistics’ Australian Statistical Geography Standard framework [35] and population size. Responses to sociodemographic and clinical characteristic items were recoded to discrete variables as shown in Table 1, with missing data categorised into a ‘missing’ group. Aboriginal and/or Torres Strait Islander background was coded yes or no. The number of treatment modalities was counted based on whether respondents had received surgery, radiotherapy, chemotherapy, or hormonal therapy (possible range 1–4). For this study, active surveillance and watchful waiting were included as a possible management strategy.

Descriptive statistics summarised respondent characteristics for the full sample and by follow-up care status. Bivariate chi-square analyses and multivariate logistic regression assessed associations between the sociodemographic and clinical characteristics and the optimal score on each follow-up care item. Logistic regression explored multivariate associations between sociodemographic and clinical characteristics and optimal follow-up experience indicator. All tests were two-sided and given the large sample size, and alpha was set at 0.01, with no adjustment for multiplicities.

## Results

Of the 10,662 patients receiving at least some of their cancer care within public hospitals in Victoria in 2018, 4998 (47%) completed the survey and of these 3335 (67%) were in follow-up care.

### Participant characteristics

Participant characteristics for the entire sample and by follow-up care status are shown in Table 1. While socioeconomic status, remoteness of follow-up centre, Indigenous status, and language spoken at home did not differ between those in and not in follow-up care, differences were found for all other characteristics and treatment variables. Respondents in follow-up care were more likely to be younger, female, to rate their health as generally good and to have breast cancer.

### Experiences of care during follow-up

Figure 1 shows variation in the proportion of respondents indicating they definitely received information across the seven survivorship care items. The highest proportions of respondents indicate they definitely received information related to the follow-up tests needed (71%) and the schedule of tests and checkups (73%). The percentages of respondents reporting optimal experiences on each of these items by sociodemographic and clinical factors are shown in Table 2. Items with the fewest participants reporting optimal experiences were receipt of information about new symptoms needing investigation (44%) and how people feel after treatment (46%). Characteristics consistently associated with receiving optimal care across these items in multivariate analyses (adjusted for all items in Table 2) included being male, reporting to be in excellent health, and having prostate or a haematological cancer. There was some variation in care experiences by socioeconomic status, with those from areas of higher socioeconomic advantage less likely to report optimal experiences relating to receiving a follow-up plan and how to get support if needed. Those diagnosed most recently were less likely to report optimal follow-up experiences (Table 2) especially in relation to receiving a follow-up care plan, information about follow-up tests, how to stay healthy, and what new symptoms need to be monitored for. Health system of follow-up care was not related to care experiences.

**Table 2:** Percentage of respondents reporting optimal experience^a^ for each follow-up care item by sociodemographic and clinical variables (note *p*-values multivariable^b^ Chi square tests) (*n* = 3335)

	*N*	Receive written follow-up care plan	Receive information about the follow-up tests needed	Receive information about how to stay healthy	Receive information about what new symptoms to monitor for	Receive information about how people feel emotionally after treatment	Receive information about how to get extra support if needed	Receive information about how often need tests/check-ups
		%	%	%	%	%	%	%
Total sample								
All	3335	50	71	64	44	46	50	73
Gender								
Male	1573	53	76	68	47	50	52	76
Female	1727	46	66	61	42	42	48	70
Missing	35	60	83	71	46	46	60	77
Multivariate p-value		0.012	< 0.001	< 0.001	0.011	< 0.001	< 0.001	0.019
Age group (years)								
< 50	419	41	63	58	42	43	46	66
50–59	573	51	72	68	47	45	52	74
60–69	1016	48	72	66	45	48	50	75
70–79	885	54	75	65	44	46	49	76
80 +	298	50	67	58	46	44	50	66
Missing	144	53	69	63	40	44	51	69
Multivariate p-value		0.004	0.018	0.06	0.83	0.46	0.50	0.004
Socioeconomic disadvantage^c^								
Highest disadvantage (40%)	1039	53	73	66	49	47	54	75
Mid (40%)	1169	50	71	64	43	46	49	73
Highest advantage (20%)	546	48	68	63	44	49	47	71
Missing	581	45	70	62	40	41	46	71
Multivariate p-value		0.001	0.40	0.23	0.003	0.054	0.006	0.41
Follow-up care treatment centre location^d^								
Metropolitan area	2501	49	70	63	44	45	48	72
Regional centre	686	51	75	68	48	50	56	77
Large/medium/small/remote towns	148	55	72	61	43	47	53	76
Multivariate p-value		0.80	0.20	0.11	0.17	0.046	0.030	0.08
Language spoken at home								
Not English	327	57	70	67	50	52	50	70
English	3008	49	71	64	44	45	50	73
Multivariate p-value		0.001	0.51	0.08	0.003	0.001	0.22	0.93
Aboriginal or Torres Strait Islander								
Yes	26	62	73	65	50	50	58	73
No	3309	50	71	64	44	46	50	73
Multivariate p-value		0.35	0.84	0.82	0.94	0.95	0.69	0.68
Self-reported health status								
Excellent	390	59	77	71	54	57	59	79
Good	2254	50	73	65	45	46	50	75
Fair or poor	615	43	61	58	39	37	43	64
Missing	76	47	63	58	36	42	51	67
Multivariate p-value		< 0.001	< 0.001	< 0.001	< 0.001	< 0.001	< 0.001	< 0.001
Cancer Type								
Colorectal	332	50	72	61	39	37	43	73
Breast	666	48	67	69	43	49	56	69
Prostate	369	60	80	70	45	55	57	79
Blood	607	53	77	68	53	54	56	80
Lung/Mesothelioma	183	50	70	67	45	44	50	74
Melanoma	149	44	67	48	40	33	34	75
Gyneacological^e^	156	47	62	58	47	40	49	76
Urological	217	44	71	55	36	32	33	70
Other	656	45	67	62	43	43	46	67
Multivariate p-value		0.003	0.103	< 0.001	< 0.001	< 0.001	< 0.001	0.017
Year diagnosed								
2015 or earlier	388	54	76	65	53	53	54	80
2016	171	49	73	61	49	48	53	79
2017	684	58	77	71	47	49	52	77
2018	1844	46	68	62	41	43	48	71
Missing	248	50	67	60	44	42	46	67
Multivariate p-value		< 0.001	< 0.001	0.005	0.003	0.15	0.43	< 0.001
Number of treatment modalities^f^								
1	1601	52	73	61	43	42	45	75
2	1003	49	71	66	46	48	52	73
3	557	46	69	68	47	52	57	69
4	174	46	66	70	39	51	57	70
Multivariate p-value		0.17	0.88	0.18	0.19	< 0.001	< 0.001	0.48
Health system for follow-up care^g^								
Public	2477	50	71	65	44	46	50	73
Private	613	47	71	63	46	45	49	74
Missing	245	52	71	63	42	47	49	73
Multivariate p-value		0.45	0.98	0.71	0.49	0.63	0.92	0.78

^a^Optimal care indicated by ‘Yes definitely’ response to the item. Percentage rounded to nearest whole number with decimal values ≥ 0.5 rounded up

^b^Multivariable analyses included all variables shown in the table and involved logistic regression analyses (see supplementary Table 1 for results from these analyses)

^c^Index of Relative Socioeconomic Advantage and Disadvantage [33]

^d^Based on Modified Monash Model [34]

^e^Gynaecological includes uterus, ovarian, cervical, vulvar, vaginal, endometrial, and fallopian tube

^f^Active surveillance/watchful waiting included as a management modality

^g^See text for explanation of health systems

### Variation in optimal survivorship care

The average score for the combined survivorship care experience measure was 1.21 (SD = 0.67), suggesting follow-up care was on average lower than optimal. Only 27% scored 2 indicating optimal scores on all seven items. Forty percent had scores ≥ 1.5 on this scale forming the optimal follow-up care group. In bivariate analyses, optimal follow-up care was related to gender (*p* < 0.001), socioeconomic status (*p* < 0.001), language spoken at home (*p* < 0.001), self-reported health status (*p* < 0.001), cancer type (*p* < 0.001), number of treatment modalities (*p* < 0.01), and diagnosis year (*p* < 0.01) (Table 3). These associations generally held in the multivariate analyses. The multivariable analyses suggest that women, respondents from areas of higher socioeconomic advantage, those speaking English at home, those with poor self-reported health, and those diagnosed more recently were less likely to report optimal follow-up care (Table 3). Participants diagnosed with breast, prostate, and haematological cancers were more likely than those with colorectal cancer to report optimal follow-up care.

**Table 3:** Descriptive statistics for the optimal follow-up care^a^ by sociodemographic and clinical characteristics and multivariate logistic regression results (all analyses based on sample included in multivariable regression: *n* = 3335). Adjusted odds ratio (aOR) (and 95% confidence intervals (CIs)) for demographic, disease, and treatment factors associations with optimal follow-up care

		*N*	% reporting optimal care	*Bivariate p-value*	aOR	95% CIs	*Multivariate p-value*
Total			40%				
Gender	Male	1573	45%		1		
	Female	1727	37%		0.60	0.50–0.73	
	Missing	35	43%	< *0.001*	1.25	0.58–2.67	< *0.001*
Age group	< 50	419	39%		1		
	50–59	573	42%		1.07	0.81–1.39	
	60–69	1016	41%		1.05	0.82–1.35	
	70–79	885	41%		1.06	0.82–1.36	
	80 +	298	36%		0.95	0.68–1.32	
	Missing	144	40%	*0.58*	1.29	0.84–1.98	*0.86*
Socio-economic disadvantage^b^	Highest disadvantage (40%)	1039	45%		1		
	Mid (40%)	1169	39%		0.75	0.62–0.90	
	Highest advantage (20%)	546	39%		0.71	0.56–0.90	
	Missing	581	36%	*0.001*	0.61	0.48–0.77	< *0.001*
Follow-up care treatment centre location^c^	Metropolitan area	2501	40%		1		
	Regional centre	686	44%		1.15	0.94–1.39	
	Large/medium/small/remote towns	148	41%	*0.14*	1.02	0.71–1.48	*0.38*
Language spoken at home	Not English	327	48%		1		
	English	3008	40%	*0.001*	0.63	0.49–0.80	< *0.001*
Aboriginal or Torres Strait Islander	Yes	26	50%		1		
	No	3309	40%	*0.32*	0.82	0.37–1.83	*0.62*
Self-reported health status	Excellent	390	51%		1		
	Good	2254	41%		0.62	0.50–0.78	
	Fair or poor	615	34%		0.42	0.32–0.55	
	Missing	76	33%	< *0.001*	0.36	0.20–0.63	< *0.001*
Cancer type	Colorectal	332	34%		1		
	Breast	666	43%		1.75	1.27–2.41	
	Prostate	369	47%		1.48	1.06–2.05	
	Blood	607	50%		1.93	1.42–2.61	
	Lung/Mesothelioma	183	52%		1.64	1.11–2.42	
	Melanoma	149	27%		0.84	0.54–1.31	
	Gyneacological^d^	156	35%		1.45	0.94–2.22	
	Urological	217	30%		0.83	0.56–1.22	
	Other	656	36%	< *0.001*	1.10	0.82–1.47	< *0.001*
Year diagnosed	2015 or earlier	388	50%		1		
	2016	171	46%		0.86	0.59–1.24	
	2017	684	45%		0.85	0.65–1.11	
	2018	1844	37%		0.62	0.48–0.79	
	Missing	248	38%	< *0.01*	0.69	0.49–0.98	< *0.001*
Number of treatment modalities^e^	1	1601	37%		1		
	2	1003	43%		1.28	1.07–1.52	
	3	557	45%		1.44	1.14–1.82	
	4	174	43%	< *0.01*	1.33	0.90–1.96	*0.01*
Health system for follow-up care^f^	Public	2477	41%		1		
	Private	613	39%		0.93	0.77–1.13	
	Missing	245	40%	*0.65*	1.02	0.76–1.36	*0.76*

^a^Optimal follow-up care determined from mean of seven follow-up care items (scored 0–2) with scores ≥ 1.5 indicating optimal care

^b^Based on Australian Bureau of Statistics Index of Relative Socioeconomic Advantage and Disadvantage [33]

^c^Based on Modified Monash Model location index [34]

^d^Gynaecological includes uterus, ovarian, cervical, vulvar, vaginal, endometrial, and fallopian tube

^e^Active surveillance/watchful waiting included as a management modality

^f^See text for explanation of health system

## Discussion

Data from a statewide survey of care experiences for people attending a public hospital for at least part of their cancer care provided a unique opportunity to explore survivorship care experiences for a large cohort of patients with multiple cancer types, treated with a variety of treatment modalities across a range of health settings. Survivorship care experiences varied and while in general around 50% of people reported receiving information on each of the follow-up care items, when considering the items in combination, only around 40% were assessed as receiving optimal survivorship care. Those less likely to report optimal survivorship care were female, from areas with higher socioeconomic advantage, reporting their health to be good or fair/poor, not diagnosed with breast, prostate, lung or a haematological cancer, and diagnosed more recently.

With the number of cancer survivors increasing worldwide, there is growing recognition of the importance of delivering follow-up or survivorship care that meets the medical and psychosocial needs of survivors. To this end, a number of survivorship care frameworks or recommendations have now been developed to assist health services to develop their survivorship care programmes and ensure that the dimensions of care that health professionals and survivors consider important are recognised [5, 6, 9, 11, 36]. Work assessing survivors’ experiences of follow-up care can help to identify the extent to which these frameworks are informing survivorship care. Ours is one of the few studies to have examined this issue for an Australian population of cancer survivors. Although our findings that Australian survivors were less likely to receive information regarding emotional responses to cancer and support services than information about follow-up tests is similar to results found in the US literature [26, 37, 38], the proportions reporting receiving emotional and support information were higher in our study compared to the US. While this may reflect differences in study questions, it might also reflect different practices. Although at the time of our study there were no Australian specific frameworks for survivorship care, Australia had in place a set of recommendations for optimal cancer care that provided recommendations for follow-up care that extended beyond a schedule for follow-up tests and appointments [39]. Additionally, between 2011 and 2019, the Victorian Department of Health delivered a grants programme that aimed to improve survivorship care. Since then, the Department has funded other statewide improvement work, partly with the goal to increase awareness of the need for optimal survivorship care. This activity may have helped to increase delivery of supportive care information as part of routine survivorship care in Victoria. Nonetheless, with only 40% of participants in our study reporting optimal survivorship care overall, and with some groups experiencing better care than others, the current study suggests the structures and processes in place within the health care system at the time of the survey may not be working for all Victorian patients. The survey was repeated in 2023, and we plan to analyse this dataset to assess change in the delivery of survivorship care over time.

Others have found that SES, cancer type, age, and gender influence experience of care [25, 27] although the patterns of association in these studies differ from ours. In our study using an area-based indicator of socioeconomic advantage/disadvantage, we found that those with greater disadvantage were more likely to report optimal follow-up care. Ways of assessing SES may be important in determining whether it is associated with care experiences. For instance, a study from the US involving 1320 cancer survivors used multiple indicators to determine SES including health insurance status (public, private, or uninsured), family income, and education and found that lower SES groups were less likely to report having follow-up care discussions with their health care team [27]. However, a smaller study focusing on rural cancer survivors in the US found an inverse association with quality patient provider communication and education levels and income level [40]. An Australian study that also used an area level indicator of SES similar to the one used in our study, found no association between SES and patient communication, involvement, and provision of supportive services during cancer care [41]. More work is needed to explore the association between SES and care experiences. While we speculate that the pattern of associations we found may reflect differences in expectations or priorities, research is needed to identify the factors underlying our results.

Although others have found that people in rural and remote areas experience significant disparities in access to cancer treatment [42], our study did not find a significant association between follow-up care experiences and location of treatment service. Another Australian study also found no difference in the experiences of information provision and patient involvement during cancer care for people living in metropolitan and rural/remote areas [41]. These are positive findings that suggest survivors experience similar quality of follow-up care in metropolitan and regional/rural health services.

Our study found that males were more likely to report optimal survivorship experiences than females. While some other studies have also found that females are more likely to report poor care experiences [29, 43], others have not [27, 40]. A large population-based Australian study looking at communication throughout cancer care and provision of information and services found no difference in the experiences of males and females [41]. There are limited studies to explain the differences in care experiences by gender. One study found females reported higher need and regard for psychosocial support than men, which may explain differences [44]. This study suggested that men are more likely to receive support from sources external to the health system and hence may rate their experiences within the health system as more positive [44]. Other work has noted that patients with breast, melanoma, haematological, and testicular cancers are less likely to make negative comments about their care, whereas patients with small intestine/rarer lower gastrointestinal, hepatobiliary, and renal cancers were most likely to report negative experiences [43]. Our results align with this pattern for breast and haematological cancers but not melanoma. While more work is needed to confirm our findings for gender, if found to be true in other Australian populations, the factors influencing poorer survivorship care experiences for women with cancers other than breast need to be investigated. Our findings relating to differences in survivorship care experiences for different cancer types also suggest more work is needed to understand reasons for this.

Those reporting to have better health were more likely to report optimal survivorship care. While our results need to be confirmed, reasons for these differences need to be explored. Our results may reflect that those with better health have simpler health needs and are more satisfied with the information they receive. Those with poor self-reported health were less likely to report optimal care across all seven follow-up care items. We do not know if the poorer state of health is due to their cancer or to other health conditions. If the latter, our findings may reflect poor integration of care across different health conditions. If the former, it may reflect the greater needs of this group of survivors. While work to understand these differences is needed, our findings suggest that health services could direct further intervention efforts to those who rate their own health as poor.

## Strengths and limitations

Principal strengths of this study are its large sample size and inclusion of participants from a range of health services and with a range of cancers. However, a number of limitations need to be noted. While many people in Australia have their cancer treatment in both the public and private systems, this study identified patients for inclusion through public hospitals only. Therefore, people who received all their cancer care in the private system were not included. Socioeconomic status was inferred by postcode, which may not be as reliable as a person-level indicators such as income, education, or occupation status. While we included treatment and cancer type in our multivariable models, we did not have information on disease stage, health service utilisation, or treatment outcomes and hence could not control for their potential impact. As data were self-reported, bias in recall due to the time since people entered follow-up may influence findings.

Although the survey was available in a variety of languages, representation of people from culturally and linguistically diverse backgrounds, as indicated by language spoken at home, was relatively low. Further research to enhance inclusivity of these populations is required. There was a relatively low response rate of 47%, which places the results at risk of selection bias. However, the response rate is comparable to other surveys of patient experiences conducted in Australia [45, 46]. Our study also had a low number of responses from Aboriginal and Torres Strait Islander peoples, which precluded undertaking analysis of this data. Strategies to engage and support Aboriginal and Torres Strait Islander peoples to participate in studies to assess their care experiences, including using culturally appropriate questions, are needed [47, 48].

## Conclusion

Large numbers of Australian cancer survivors report sub-optimal survivorship care. Further work is needed to understand the factors influencing survivorship care experiences for different groups of patients, particularly for women with cancers other than breast and for those with poor self-reported health. Ongoing monitoring of patients’ care experiences during and post-treatment can provide important information relating to the quality of care and gaps in services. Our findings suggest that further work is needed to increase the provision of information to survivors about new symptoms to monitor for, common emotional responses after treatment, and ways to access support when needed.

## Supplementary Information

Below is the link to the electronic supplementary material.

Supplementary file1 (XLSX 28 KB)

## Data Availability

The data that support the findings of this study are available from the Victorian Agency for Health Information, Melbourne, Victoria, Australia. Restrictions apply to the availability of these data, which were used under license for this study.
